# Wavelet-Based Identification for Spinning Projectile with Gasodynamic Control Aerodynamic Coefficients Determination

**DOI:** 10.3390/s22114090

**Published:** 2022-05-27

**Authors:** Piotr Lichota, Mariusz Jacewicz, Robert Głębocki, Dariusz Miedziński

**Affiliations:** Institute of Aeronautics and Applied Mechanics, Warsaw University of Technology, 00-665 Warsaw, Poland; mariusz.jacewicz@pw.edu.pl (M.J.); robert.glebocki@pw.edu.pl (R.G.); dariusz.miedzinski2.dokt@pw.edu.pl (D.M.)

**Keywords:** aerodynamics, flight dynamics, system identification, wavelet transform

## Abstract

Identification of a spinning projectile controlled with gasodynamic engines is shown in this paper. A missile model with a measurement inertial unit was developed from Newton’s law of motion and its aerodynamic coefficients were identified. This was achieved by applying the maximum likelihood principle in the wavelet domain. To assess the results, this was also performed in the time domain. The outcomes were obtained for two cases: when noise was not present and when it was included in the data. In all cases, the identification was performed in the passive mode, i.e., no special system identification experiments were designed. In the noise-free case, aerodynamic coefficients were estimated with high accuracy. When noise was included in the data, the wavelet-based estimates had a drop in their accuracy, but were still very accurate, whereas for the time domain approach the estimates were considered inaccurate.

## 1. Introduction

System identification is a process in which a mathematical model of the investigated object is obtained from input and output signal measurements. This approach is widely used in aeronautical engineering to obtain aerodynamic derivatives as it deals with the true object and thus allows validation of outcomes from wind tunnel tests or computational fluid dynamics [1].

The quality of the performed system identification depends on the amount of information that is stored in the measured output signals [2]. Thus, it also depends on the input signals, and usually special system identification experiments are designed. In those experiments, either an engineering-based approach with multi-step [3,4], multisine [5,6] or frequency sweep [7,8] inputs is used, or the inputs are designed with optimality criteria [9,10]. This approach is known as active system identification. It is also possible to perform the system identification process from the output signals registered when the system is normally operating, provided that the amount of information stored in the measurements is rich and diverse enough [11]. This approach is known as passive system identification. It is rarely used in aeronautical engineering as in scheduled operations the flight parameters are almost constant or slowly change during the nonterminal flight phases, whilst during take-off or landing air traffic control commands force specific pilot reactions (which also limits the amount of diverse information stored in the measurements). However, this approach can be useful, e.g., in missile system identification, especially when the number of projectiles and their launches is limited.

When dealing with engines controlled with gasodynamic inputs, this approach is also useful because of the limited control capabilities as each engine is simply launched and burns out and thus it is hard or not possible to execute a complex control strategy [12]. Moreover, if the aircraft were rotating along the longitudinal axis at a significant rate, the conventional aircraft system identification inputs or optimal excitations would have even stronger design limitations. Thus, it was decided to perform the spinning projectile controlled with gasodynamic engine identification from a missile flight for which the inputs were not designed, i.e., in passive mode.

In the study, the missile was to hit the aim (reference model); the trajectory was evaluated earlier and in the control algorithm the flight data directly from the equations of motion or from the Inertial Measurement System (IMU) were used. When the missile is rotating at a high rate and medium-cost sensors are selected, the measurement errors can significantly lower the estimation quality when the equations of motion are integrated, as the flight parameters at each point depend on their values at the previous point. Thus, it was not possible to use the most popular system identification approach—the time domain output error method. However, this would not be a problem for fixed wing aircraft, helicopters, or non-spinning missiles, as the changes in their attitude occur at a slower rate and it would be possible to use this approach. On the other hand, the missile under study was flying at high Mach numbers and thus the compressibility effects had to be included. Due to the nonlinearities, it was not possible to use system identification frequency domain methods as well.

Thus, a different approach was applied—the wavelet domain was used to perform the parameter estimation as it allows one to discard the noise components in the data and still deal with the nonlinear object. This is done by decomposing the flight parameters into coefficients; the values change in relation to their frequency and their time localization [13]. This is similar to Fourier transform and is used in aeronautics for fault diagnostics [14,15], detecting manoeuvres [16], system identification experiment design [9,17] and physical phenomena prediction [18,19].

Investigating the possibility of mixing the wavelet domain with the maximum likelihood principle to perform passive system identification was the aim of this study and is the main novelty of this paper. To investigate and develop this method, a simulation model was used and it was treated like a real object. This approach is common in flight dynamics [20,21] as it allows one to limit costs when new methods or aircraft modifications are designed.

The organization of this paper is as follows. A short introduction is presented in this part (Section 1), followed by the object description and its mathematical formulation, shown in Section 2. Then, in the Section 3, the maximum likelihood approach with wavelet-based domain identification is presented. In the next part (Section 4) the results are presented for the noise-free case when using the wavelet-based approach. The time domain output error method outcomes are also shown to compare those approaches under ideal conditions (Section 4.1 and Section 4.2, respectively). Then, the same is done for measurement noise presence in Section 4.3 and Section 4.4. The paper finishes with a short summary of conclusions in Section 5.

## 2. Model

The object used in the study was a spinning missile which had a main engine that was used to launch the rocket and 32 gasodynamic thrusters used for control. The thrusters were placed radially in four layers (eight in each) due to missile-limited dimensions (0.122 m diameter). Additionally, each layer was rotated 11.25 degrees, so all thrusters were evenly spaced along the missile circumference to extend the control capabilities. It was possible to launch each engine once and during its work it produced an 800 N thrust that lasted for 30 ms.

The missile was axisymetric and modelled as a rigid body in the vehicle-carried coordinate system Oxyz. The Ox axis coincided with the missile longitudinal axis and was directed towards the missile nose cone. The Oy axis pointed towards the right stabilizer and the Oz axis completed the right-handed coordinate system.

The equations of motion were developed from the momentum and angular momentum change theorems in a moving reference frame:

(1) $$\begin{array}{c} \frac{\tilde{\delta }\Pi }{\tilde{\delta }t}+\Omega \times \Pi =F\\ \frac{\tilde{\delta }K_{O}}{\tilde{\delta }t}+\Omega \times K_{O}+V\times \Pi =M_{O} \end{array}$$

where V and Ω are the linear and angular velocity, Π and $K_{O}$ stand for momentum and angular momentum, F and $M_{O}$ are external forces and moments and $\frac{\tilde{\delta }}{\tilde{\delta }t}$ denotes the local derivative.

Momentum and angular momentum were defined for the rigid body model:

(2) $$\begin{array}{c} \Pi =m\left(V+\Omega \times r_{C}\right)\\ K_{O}=I\Omega +r_{C}\times mV \end{array}$$

where *m* is the rocket mass, I denotes the inertia matrix and $r_{C}$ is the centre of gravity offset.

The external forces and moments resulted from aerodynamics, propulsive thrusters and gravity.

The aerodynamic components were evaluated in an aerodynamic experimental frame and thus it was necessary to take into account the offset between its origin ($O_{e}$) and the origin of the vehicle-carried system Oxyz.

(3) $$\begin{array}{c} F_{a}=\frac{1}{2}\rho {\left|V\right|}^{2}S\left[\begin{array}{c} C_{X}\\ C_{Y}\\ C_{Z} \end{array}\right]\\ M_{a,O_{e}}=\frac{1}{2}\rho {\left|V\right|}^{2}Sd\left[\begin{array}{c} C_{l}\\ C_{m}\\ C_{n} \end{array}\right]+r_{e}\times F_{a} \end{array}$$

where *d* is the diameter, *S* is the cross section area, $r_{e}$ is the experimental frame offset. The aerodynamic coefficients were obtained from Prodas software and can be expressed as:

(4) $$\begin{array}{c} C_{X}=\left(C_{X_{base\;0}}+C_{X_{base\;\alpha^{2}}}\alpha^{2}+C_{X_{base\;\beta^{2}}}\beta^{2}\right)+\left(C_{X_{eng\;0}}+C_{X_{eng\;\alpha^{2}}}\alpha^{2}+C_{X_{eng\;\beta^{2}}}\beta^{2}\right)\delta_{eng}\\ C_{Y}=C_{Y_{0}}+C_{Y_{\beta }}\beta \\ C_{Z}=C_{Z_{0}}+C_{Z_{\alpha }}\alpha \\ C_{l}=C_{l_{0}}+\left(C_{l_{p_{0}}}+C_{l_{p_{\alpha^{2}}}}\alpha^{2}+C_{l_{p_{\beta^{2}}}}\beta^{2}\right)\frac{pd}{2|V|}\\ C_{m}=C_{m_{0}}+C_{m_{\alpha }}\alpha \\ C_{n}=C_{n_{0}}+C_{n_{\beta }}\beta \end{array}$$

where α and β are the angle of attack and the sideslip angles, *p* is the roll rate and $\delta_{eng}$ is the main engine control.

The propulsive forces resulting from the main engine thrust and the corresponding moment were evaluated as:

(5) $$\begin{array}{c} F_{\mathrm{prop}}=F_{eng}\left(t\right)\left[\begin{array}{c} \cos\Theta_{T}\cos\Psi_{T}\\ \cos\Theta_{T}\sin\Psi_{T}\\ -\sin\Psi_{T} \end{array}\right]\\ M_{\mathrm{prop}}=r_{\mathrm{eng}}\times F_{\mathrm{prop}} \end{array}$$

where $F_{eng}$ is the engine thrust, $\Theta_{T}$ and $\Psi_{T}$ are the thrust force deflection angles and $r_{\mathrm{eng}}$ is the engine offset.

The total force and moment resulting from the gasodynamic engine control were given as:

(6) $$\begin{array}{c} F_{\mathrm{cmd}}=\sum_{i=1}^{M}\sum_{j=1}^{N}F_{th_{i,j}}\left[\begin{array}{c} 0\\ \sin\Phi_{i,j}\\ -\cos\Phi_{i,j} \end{array}\right]\\ M_{\mathrm{cmd}}=\sum_{i=1}^{M}\sum_{j=1}^{N}r_{{\mathrm{cmd}}_{i,j}}\times F_{th_{i,j}} \end{array}$$

where i=1,⋯,M and j=1,⋯,N are the gasodynamic engine layer and engine number in each layer, $F_{th_{i,j}}$ is the single gasodynamic engine thrust, $r_{{\mathrm{cmd}}_{i,j}}$ is the engine offset and $\Phi_{i,j}$ is its angular position.

Finally, the moment due to gravity is given as:

(7) $$M_{g}=r_{C}\times F_{g}$$

where the gravity force $F_{g}$ is given in the vehicle-carried axes.

As the object can be launched at 90 degrees, the quaternion algebra was used for attitude determination to avoid singularities and modelling errors. The quaternion kinematic equation was given as:

(8) $$\left[\begin{array}{c} {\dot{e}}_{0}\\ {\dot{e}}_{1}\\ {\dot{e}}_{2}\\ {\dot{e}}_{3} \end{array}\right]=-\frac{1}{2}\left[\begin{array}{cccc} 0 & P & Q & R\\ -P & 0 & -R & Q\\ -Q & R & 0 & -P\\ -R & -Q & P & 0 \end{array}\right]\left[\begin{array}{c} e_{0}\\ e_{1}\\ e_{2}\\ e_{3} \end{array}\right]-kE\left[\begin{array}{c} e_{0}\\ e_{1}\\ e_{2}\\ e_{3} \end{array}\right]$$

where $e_{0}$, $e_{1}$, $e_{2}$ and $e_{3}$ are quaternion components, whilst *k* and *E* are the feedback coefficient and bounding equation violation coefficient. After integration, it was possible to evaluate the missile attitude:

(9) $$\begin{array}{c} \Phi =\arctan\frac{2(e_{0}e_{1}+e_{2}e_{3})}{e_{0}^{2}-e_{1}^{2}-e_{2}^{2}+e_{3}^{2}}\\ \Theta =\arcsin2(e_{0}e_{2}-e_{1}e_{3})\\ \Psi =\arctan\frac{2(e_{0}e_{3}+e_{1}e_{2})}{e_{0}^{2}+e_{1}^{2}-e_{2}^{2}-e_{3}^{2}} \end{array}$$

where Φ, Θ and Ψ are roll, pitch and yaw angles, respectively.

As mentioned, the inertial measurement system was also included in the model. The system consisted of a three-axis accelerometer with a *g* offset and three-axis rate gyro. As the sensors were not mounted at the origin of the reference frame, the acceleration recorded at a specific point was evaluated as:

(10) $$a_{IMU}=a+\Omega \times \left(\Omega \times r_{IMU}\right)+\dot{\Omega }\times r_{IMU}-g$$

where a=F/m is the evaluated acceleration, $r_{IMU}$ is the IMU offset and the dot symbol denotes the derivative with respect to time.

Moreover, for the accelerometers, the mounting errors and bias were taken into account:

(11) $${\hat{a}}_{IMU}=\left[\begin{array}{ccc} s_{acc_{x}} & -c_{acc_{xy}} & c_{acc_{xz}}\\ c_{acc_{xy}} & s_{acc_{y}} & -c_{acc_{yz}}\\ -c_{acc_{xz}} & c_{acc_{yz}} & s_{acc_{z}} \end{array}\right]a_{IMU}+\left[\begin{array}{c} b_{acc_{x}}\\ b_{acc_{y}}\\ b_{acc_{z}} \end{array}\right]$$

where $s_{acc_{x}}$, $s_{acc_{y}}$, $s_{acc_{z}}$ are the accelerometer scale factors, $c_{acc_{xy}},c_{acc_{xz}},c_{acc_{yz}}$ are the misaligment factors, and $b_{acc_{x}},b_{acc_{y}},b_{acc_{z}}$ are biases.

For the gyroscopes, additionally the G-sensitivity was included:

(12) $${\hat{\Omega }}_{IMU}=\left[\begin{array}{ccc} s_{gyro_{x}} & -c_{gyro_{xy}} & c_{gyro_{xz}}\\ c_{gyro_{xy}} & s_{gyro_{y}} & -c_{gyro_{yz}}\\ -c_{gyro_{xz}} & c_{gyro_{yz}} & s_{gyro_{z}} \end{array}\right]\Omega_{IMU}+\left[\begin{array}{c} b_{gyro_{x}}\\ b_{gyro_{y}}\\ b_{gyro_{z}} \end{array}\right]+G\left[\begin{array}{c} a_{x}\\ a_{y}\\ a_{z} \end{array}\right]$$

where $s_{gyro_{x}}$, $s_{gyro_{y}}$, $s_{gyro_{z}}$ are the gyroelerometer scale factors, $c_{gyro_{xy}},c_{gyro_{xz}},c_{gyro_{yz}}$ are the misaligment factors, $b_{gyro_{x}},b_{gyro_{y}},b_{gyro_{z}}$ are biases and *G* is the G-sensitivity matrix.

To include the IMU dynamics, all sensors were treated as a second-order system:

(13) $$\begin{array}{c} a_{meas}=\frac{\omega_{n_{acc}}^{2}}{s^{2}+2\xi_{acc}\omega_{n_{acc}}s+\omega_{n_{acc}}^{2}}{\hat{a}}_{IMU}\\ \Omega_{meas}=\frac{\omega_{n_{gyro}}^{2}}{s^{2}+2\xi_{gyro}\omega_{n_{gyro}}s+\omega_{n_{gyro}}^{2}}{\hat{\Omega }}_{IMU} \end{array}$$

where $\omega_{n_{acc}}$ and $\xi_{acc}$ are the natural frequency and damping coefficient of the accelerometer, whilst $\omega_{n_{gyro}}$ and $\xi_{gyro}$ are the natural frequency and damping coefficient of the rate gyro.

The IMU measurements were also corrupted by adding a measurement noise:

(14) $$\begin{array}{c} {\hat{a}}_{meas}=a_{meas}+\nu_{acc}\\ {\hat{\Omega }}_{meas}=\Omega_{meas}+\nu_{gyro} \end{array}$$

where $\nu_{acc}$ and $\nu_{gyro}$ stand for accelerometers and rate gyro measurement noise.

**Figure 1:**
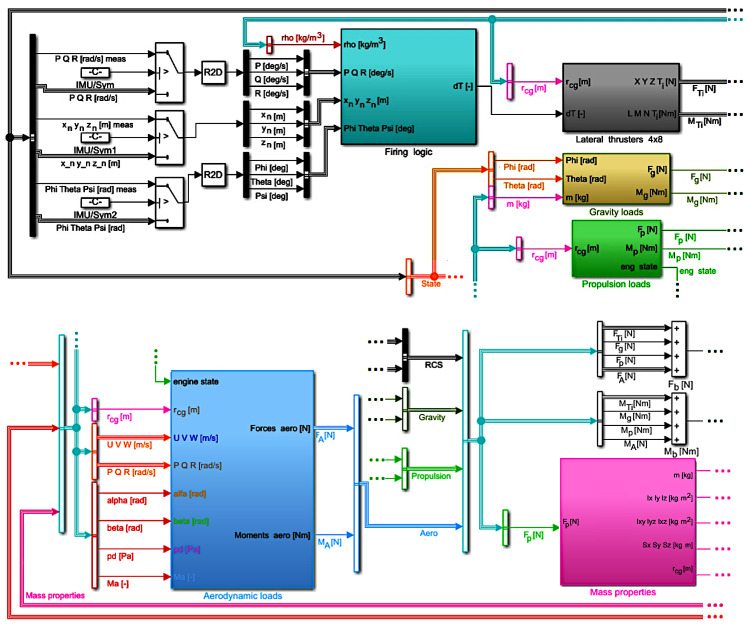

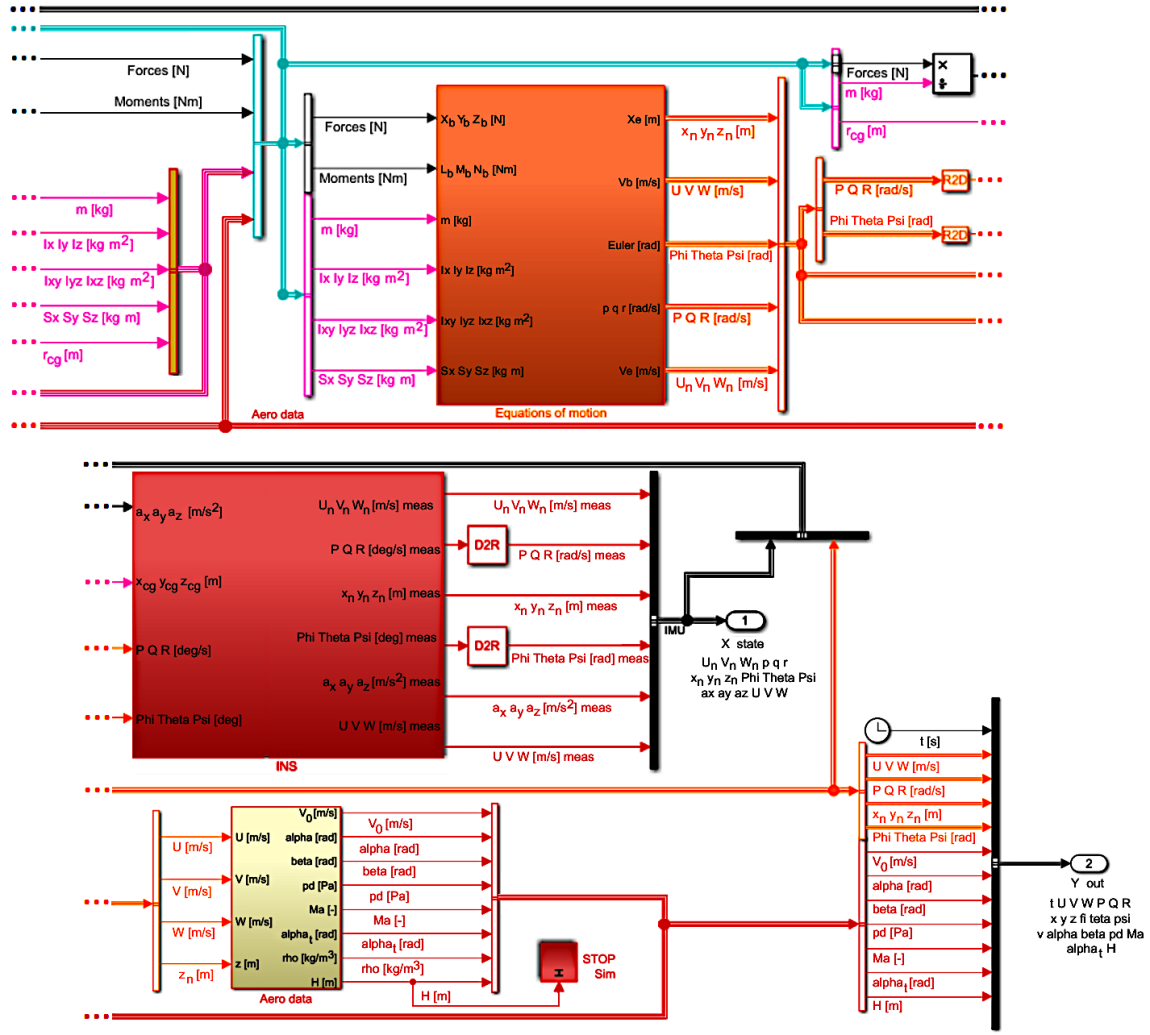
Simulation model structure—top level.

The top level architecture for the described simulation model is shown in Figure 1.

## 3. Method

The flight parameters of the spinning projectile were gathered assuming that it was normally operating, i.e., during a trajectory tracking task. The registered flight parameters were: linear and angular velocity components (*u*, *v*, *w* and *p*, *q*, *r*, respectively), aerodynamic angles (α, β), attitude angles (ϕ, θ, ψ) position in earth fixed frame ($x_{n}$, $y_{n}$, $z_{n}$), Mach number (Ma ) and altitude *H*.

**Figure 2:**
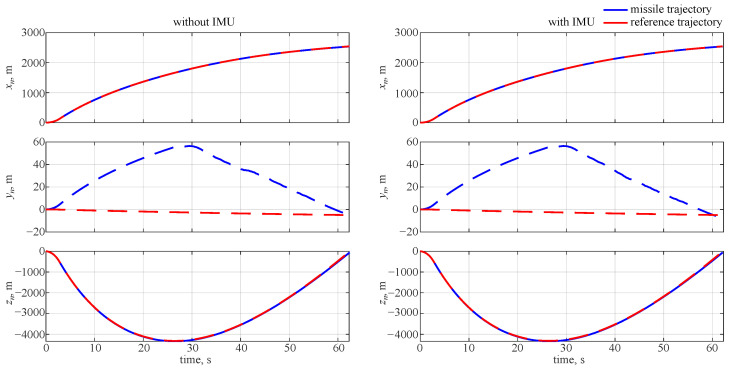
Missile and reference trajectory.

The reference trajectory and the missile position time history are shown in Figure 2 for flight parameters evaluated directly from the equations of motion or obtained from the IMU. It can be seen from the plot that an offset in the lateral position was obtained for the final position; however, this was considered acceptable as the error was of just a few meters. When the IMU was not used, i.e., assuming an ideal system, this offset was not observed.

**Figure 3:**
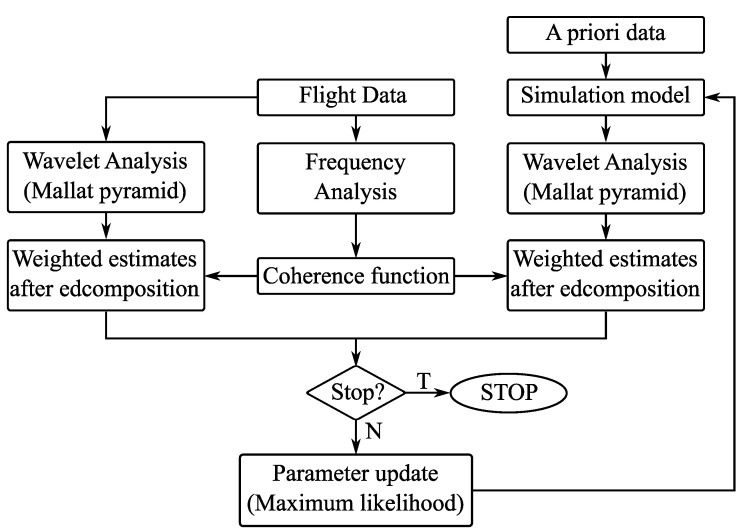
System identification flow chart.

The system identification method applied in this study is shown in Figure 3 and its components are described in the next sections.

### 3.1. Wavelet Analysis

Wavelet transform is a tool that allows decomposition of a base signal into components with specific frequencies that can be observed at a given time. This is done by using a family of functions named wavelets, and changing the values of their two parameters corresponding to frequency (scale parameter *a*) and time offset (location parameter *b*):

(15) $$\Psi_{a,b}\left(t\right)=\frac{1}{\sqrt{a}}\Psi \left(\frac{t-b}{a}\right)$$

where Ψ is the wavelet function.

To limit the computational resources needed to perform the wavelet analysis and shorten its time, the discrete wavelet transform can be applied. This can be done by using the Mallat pyramid scheme [22], which decomposes the base signal into approximations and details by passing the base signal through the low and high pass filters. Then, this process is repeated for the approximations a specified number of times. Moreover, to keep the data sample number equal, after each filter, the signals are downsampled. The approximation and detail coefficients when multiple flight parameters are decomposed are given as:

(16) $$\begin{array}{ccc} A_{l}^{i,j} & = & \sum_{k}h_{\Psi }\left[k-2l\right]A_{k}^{i,j-1}\\ D_{l}^{i,j} & = & \sum_{k}g_{\Psi }\left[k-2l\right]D_{k}^{i,j-1} \end{array}$$

where *i* is the flight parameter index, *j* is the decomposition level that corresponds to the discrete frequency, whilst $h_{\Psi }\left[2k-l\right]$ and $g_{\Psi }\left[2k-l\right]$ are the high- and low-pass filters delayed by 2l.

To perform the decomposition, the Haar wavelet was used, as it was found to be efficient when obtaining information about object dynamics from flight parameters [9]. In the study, the Haar wavelet was used for decomposition:

(17) $$\Psi \left(t\right)=\left\{\begin{array}{cc} 1 & 0\leq t<0.5\\ -1 & 0.5\leq t<1\\ 0 & t\notin [0\;1) \end{array}\right.$$

Therefore, the high- and low-pass filter coefficients were $h_{\Psi }=\left[\begin{array}{cc} 1/\sqrt{2} & 1/\sqrt{2} \end{array}\right]$ and $g_{\Psi }=\left[\begin{array}{cc} 1/\sqrt{2} & -1/\sqrt{2} \end{array}\right]$. It would be possible to use other wavelet types, e.g., Daubechies or Mayver wavelets, but this would extend the system identification time. As the later results (presented in Section 4) were found to be accurate, other wavelet types were not used in the study.

As more often it is easier to verify the outcomes based on the time history plots, the signal reconstruction formula was also incorporated in the system identification algorithm:

(18) $$y_{i}=\sum_{l}h_{\Psi }\left[k-2l\right]A_{k}^{i,j}+\sum_{j}\sum_{l}g_{\Psi }\left[k-2l\right]D_{k}^{i,j}$$

In the study, the simulation model step was 0.002 s. However, the magnitude squared coherence function for the high frequencies was below 0.6, which is assumed as a lower threshold for performing system identification [23]. Thus, it was found that no important information about the object dynamics will be lost if the data are reduced by a factor of 10, which would correspond to 0.02 s and significantly limit the time required for signal decomposition. This is a 50 Hz upper limit, which is a typical value for aircraft system identification. The gasodynamic engines were allowed to be launched around 25 s. To allow for static term estimation, an additional 5 s before the first possible engine launch was included in the data. This means that 40 s of data was used for estimation, which corresponds to *j* = 12 decomposition level.

### 3.2. Coherence Weighting

To put a greater emphasis on the data quality, a weighting function was used. The data reliability was assessed by using a magnitude squared coherence function, which is a common practice in aircraft system identification performed in the frequency domain [23]. This was done in the CIFER software by evaluating smooth spectral estimates from the time domain data transformed to the frequency domain by using the chirp-Z transform.

(19) $$\begin{array}{c} {\hat{S}}_{xx}\left(f\right)=\frac{1}{Un_{r}}\sum_{k=1}^{n_{r}}\frac{2}{T}{\left|X\left(f\right)\right|}^{2}\\ {\hat{S}}_{yy}\left(f\right)=\frac{1}{Un_{r}}\sum_{k=1}^{n_{r}}\frac{2}{T}{\left|Y\left(f\right)\right|}^{2}\\ {\hat{S}}_{xy}\left(f\right)=\frac{1}{Un_{r}}\sum_{k=1}^{n_{r}}\frac{2}{T}\left|X^{†}\left(f\right)Y\left(f\right)\right| \end{array}$$

where $S_{xx}$, $S_{yy}$ and $S_{xy}$ are the auto-spectra for inputs, outputs and the cross-spectrum, respectively. The time segment number (with 0.8 overlap) is denoted as $n_{r}$, whilst the correction factor for the half-sine window is denoted as *U*.

This leads to a a single-input single-output system magnitude squared coherence function:

(20) $${\hat{\gamma }}_{xy}^{2}\left(f\right)=\frac{|{\hat{S}}_{xy}{\left(f\right)|}^{2}}{|{\hat{S}}_{xx}\left(f\right)||{\hat{S}}_{yy}\left(f\right)|}$$

On this basis, multiple-input single-output system spectral functions are given as:

(21) $$\hat{H}\left(f\right)={\hat{S}}_{xx}^{-1}\left(f\right){\hat{S}}_{xy}\left(f\right)$$

This procedure can be performed for each output and the results can be combined, leading to the multi-input multi-output system spectral functions. In the study, additionally, the analysis was performed for five time window lengths and the results were combined by using the composite technique [23]. This results in high quality spectral function estimates at low, middle and high frequencies. Finally, the coherence function for each flight parameter is given as:

(22) $$\gamma_{xy_{c}}^{2}=\frac{|{\hat{S}}_{xy_{c}}{\left(f\right)|}^{2}}{|{\hat{S}}_{xx_{c}}\left(f\right)||{\hat{S}}_{yy_{c}}\left(f\right)|}$$

where the *c* index stands for the composite corrected spectral estimate. In aircraft modelling, it is assumed that if the coherence is below 0.6, the data should not be used in system identification. In the present study, it was found that the flight parameters below a j=6 decomposition level would not introduce significant information about the aircraft dynamics to the system identification process. Thus, for the parameter estimation, only components at *j* = 6, ..., 12 decomposition levels were used.

The weighting function that was used for adjusting the wavelet coefficients with respect to their reliability was the standard function used when identifying the frequency responses [23]:

(23) $$W_{\gamma }={\left[1.58(1-e^{-\gamma_{xy_{c}}^{2}})\right]}^{2}$$

### 3.3. Maximum Likelihood Principle

The wavelet transforms with coherence weighting were incorporated into the maximum likelihood principle that was used for parameter estimation. In this approach, a set of parameters Θ that has the greatest probability *p* of observing the measurements *z* is looked for [1]:

(24) $$\hat{\Theta }=\arg\max_{\Theta }\;p\left(z|\Theta \right).$$

In the study, the multivariate normal distribution was selected for the maximum likelihood principle as it is well suited for flight dynamic purposes. For the independent observations, it is given as:

(25) $$p\left(\tilde{z}|\Theta \right)={\left({\left(2\pi \right)}^{n}\det\left(R\right)\right)}^{-N/2}\exp\left[-\frac{1}{2}\sum_{k=1}^{N}{\left[\tilde{z}\left(k\right)-\tilde{y}\left(k\right)\right]}^{T}R^{-1}\left[\tilde{z}\left(k\right)-\tilde{y}\left(k\right)\right]\right]$$

where *y* is the model outputs, *n* and *N* are numbers of decomposed outputs and time points, *R* is the measurement noise covariance matrix, whilst the tilde symbol $\tilde{}$ denotes the decomposed time domain signal at a given decomposition level.

As the probability density is given by an exponential function, it is useful to replace it with a negative log-likelihood:

(26) $$L\left(\Theta |\tilde{z}\right)\equiv p\left(\tilde{z}|\Theta \right)$$

Thus, the cost function is:

(27) $$L\left(\Theta |\tilde{z}\right)=\frac{1}{2}\sum_{k=1}^{N}{\left[\tilde{z}\left(k\right)-\tilde{y}\left(k\right)\right]}^{T}R^{-1}\left[\tilde{z}\left(k\right)-\tilde{y}\left(k\right)\right]+\frac{nN}{2}\ln\left(2\pi \right)+\frac{N}{2}\ln\left(\det\left(R\right)\right)$$

and the problem results in a cost function minimization task.

The unknown measurement noise covariance matrix can be estimated from the residuals:

(28) $$\hat{R}=\frac{1}{N}\sum_{k=1}^{N}\left[\tilde{z}\left(k\right)-\tilde{y}\left(k\right)\right]{\left[\tilde{z}\left(k\right)-\tilde{y}\left(k\right)\right]}^{T}$$

Finally, after substituting the measurement noise covariance matrix estimate into the negative log-likelihood, omitting constant terms, and incorporating the coherence weighting function, the cost function is given as:

(29) $$J\left(\Theta \right)=\prod_{i=1}^{n}\left(W_{\gamma_{i}}\frac{1}{N}\sum_{k=1}^{N}{\left({\tilde{z}}_{i}\left(k\right)-{\tilde{y}}_{i}\left(k\right)\right)}^{2}\right)$$

To perform the parameter estimation, linear and angular velocity components were used. Unknown aerodynamic coefficient changes with respect to the Mach number were expressed as polynomials; the coefficients were to be identified. Moreover, due to the missile axial symmetry, the selected force and moment coefficients were assumed to be fixed or equal. The aerodynamic coefficient structure is presented in Table 1.

## 4. Results

### 4.1. Noise-Free Case—Wavelet-Based Approach

To check if it is possible to use the wavelet-domain approach to accurately estimate the aerodynamic derivatives for the spinning projectile controlled through gasodynamic engines, a noise-free case was investigated first. In this case, the IMU model was not used and the flight parameter changes were evaluated directly from the equations of motion. As mentioned earlier, those flight parameters were decomposed by using the Mallat Pyramid scheme and this formed the observation vector z, to which the model outputs y were fitted. This was done for a single time segment starting 5 s before the gasodynamic control was able to be used.

**Figure 4:**
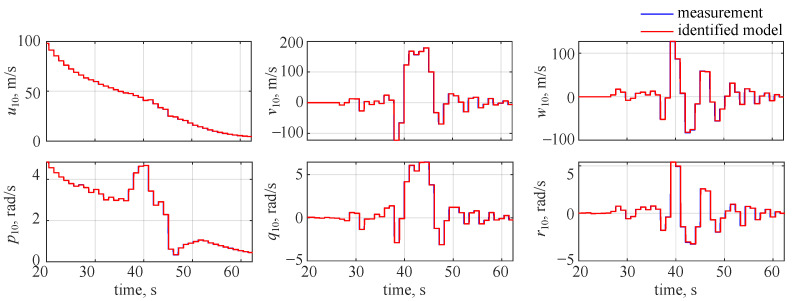
Identified wavelet coefficient time histories at the 10th decomposition level, no noise.

The observed and identified wavelet coefficients for the flight parameters at the 10th decomposition level are shown in Figure 4. The blue lines in the plot correspond to the decomposed measurements and the red lines denote the estimated outputs. An almost ideal match can be observed in the plot—the estimated outcomes (red lines) overlap with the measurements (blue lines), making them almost not visible in the plot.

**Figure 5:**
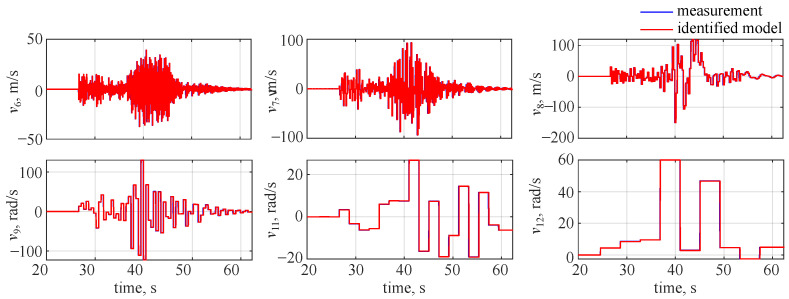
Identified side velocity wavelet coefficient time histories, no noise.

This was observed for all used decomposition levels and is shown for the lateral velocity wavelet coefficients in Figure 5. The blue lines in the plot denote the decomposed measurements and the red lines stand for the decomposed estimated flight parameters. The 10th level was not presented in that plot as it was shown earlier in Figure 4, and again estimated flight parameters are almost indistinguishable from the measurements.

**Figure 6:**
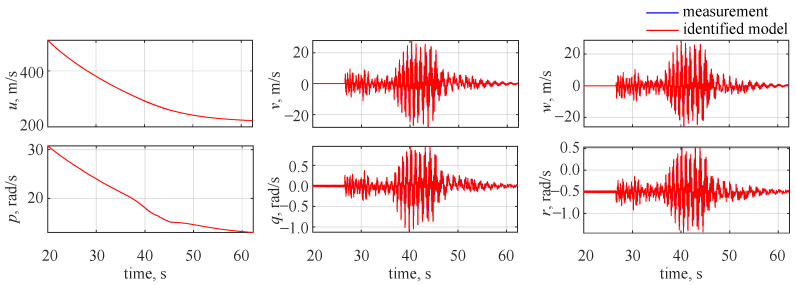
Flight parameter time histories, no noise.

On the basis of the estimated aerodynamic coefficients, it was possible to evaluate the flight parameter time histories. This is shown in Figure 6, and again an ideal visual match can be seen between the measurements and identified responses (again the estimated results overlap with the measured ones).

### 4.2. Noise-Free Case—Time Domain Approach

The noise-free dataset was used also to perform system identification when using the maximum likelihood principle formulated in the time domain, as this is the most common approach in aircraft system identification. This was aimed at finding out whether the same accuracy level can be reached and comparing the efficiency of both approaches.

As no decomposition was required, the flight parameter time histories were directly compared with the model outputs. It was found to be impossible to distinguish the estimated flight parameter time histories from the ones obtained when the wavelet-based approach was selected. Thus, the results shown in Figure 6 correspond also to the case when the system was identified in the time domain.

On the other hand, less time was required to perform system identification as there was no need for signal decomposition. In the wavelet case, it took an additional 20.7071 s per single cost function evaluation on an Intel Pentium Quad Core laptop running at 1.80 GHz with 16 GB RAM in a Windows 10 operating system. This means that the wavelet-based method for projectiles spinning at high rates cannot be applied in near real time. When high-quality sensors are used (i.e., negligible noise in the flight data), the time domain formulation should be used instead.

### 4.3. Noise Present in the Data—Wavelet-Based Approach

The wavelet-based approach was also used when IMU and noise were included in the model. The methodology and system identification settings were the same as for the noise-free case, as this allowed for estimation outcome comparison.

**Figure 7:**
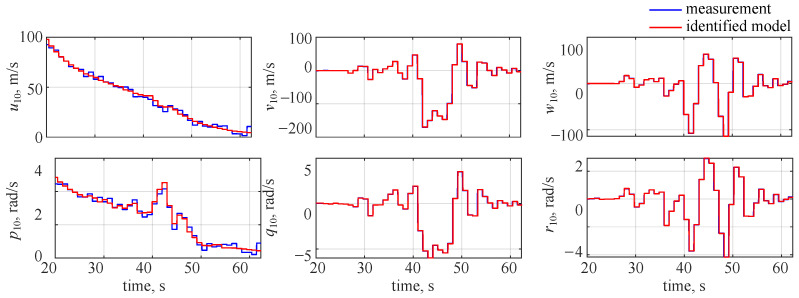
Identified wavelet coefficient time histories at the 10th decomposition level, noise included.

The identified and measured wavelet coefficients at the 10th level are shown in Figure 7, where blue lines denote the decomposed measured outputs and red lines stand for the estimated model. It can be seen that a very good visual match is observed and the only noticeable discrepancies can be seen for longitudinal velocity *u* and roll rate *p*. This comes from the fact that less variability was observed in those two flight parameters: the missile was flying at high velocity and spinning along the longitudinal axis with a significant roll rate. Thus, the reaction due to gasodynamic control was much less visible in the longitudinal velocity and roll rate.

**Figure 8:**
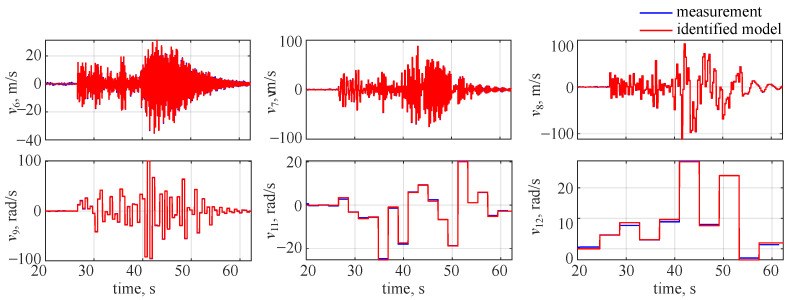
Identified side velocity wavelet coefficient time histories, noise included.

For the remaining flight parameters, an almost ideal match was observed for all decomposition levels as can be seen in Figure 8 for the lateral velocity. In this case, very small discrepancies can be noticed at the 12th decomposition level and this probably comes from the fact that the noise was averaged over a longer period.

**Figure 9:**
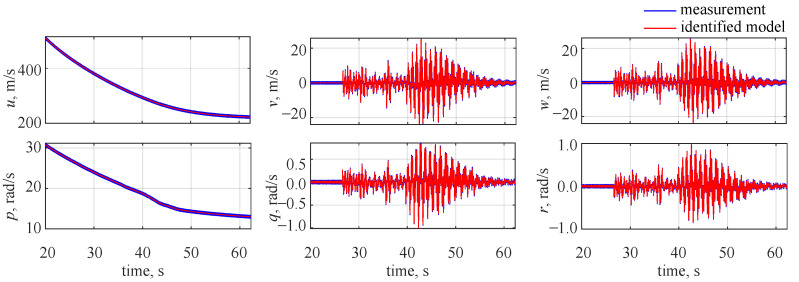
Flight parameter time histories, noise included (wavelet domain identification).

As should be expected from previous results, the flight parameter time histories matched the measured data very well. This can be observed in Figure 9 and is especially visible for the flight parameters that have distinct changes in their values due to the applied control (lateral velocity *v*, vertical velocity *w*, pitch rate *q*, roll rate *r*). However, the fit for the longitudinal velocity and roll rate is very good as well and the estimation results were just slightly worse than in the noise-free case.

**Figure 10:**
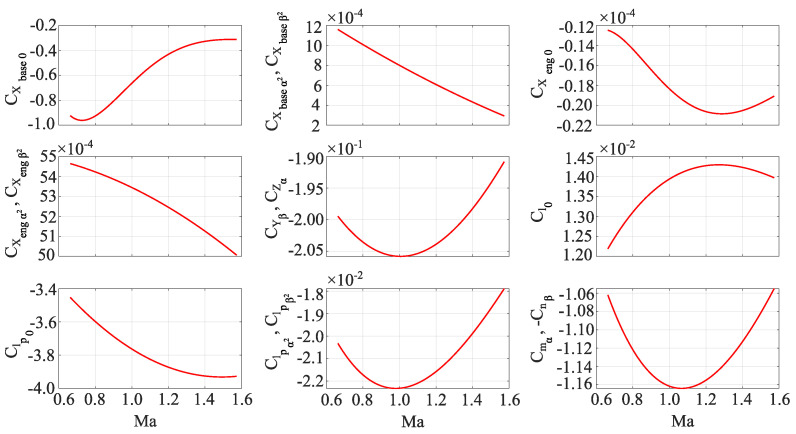
Wavelet -based identified aerodynamic coefficients, noise included.

Finally, the estimated aerodynamic coefficients as a function of their Mach number are shown in Figure 10. It can be seen that it would be possible to use lower-order polynomials for estimating some aerodynamic coefficients (engine derivatives depending on the aerodynamic angles). However, one has to have in mind that this would drop the accuracy level.

The obtained results are in good agreement with time histories, and aerodynamic coefficients presented for missiles with comparable geometrical data [12,24]. The estimates also match aerodynamic coefficients when evaluated in PRODAS in wind axes [25]. The flight parameters’ time histories are of the same type as when impulse control is used for other projectiles [26,27]. Similar results are reported also in state-of-the-art papers, e.g., [28,29,30,31].

### 4.4. Noise Present in the Data—Time Domain Approach

**Figure 11:**
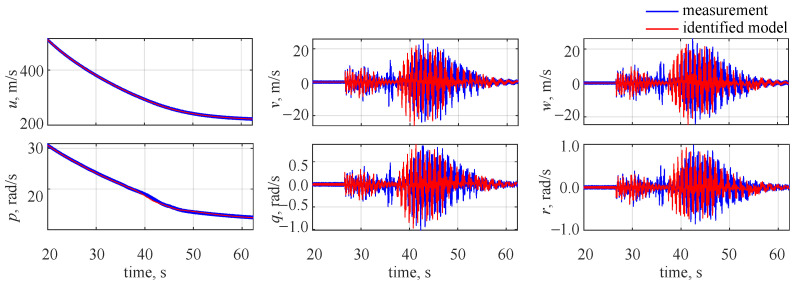
Flight parameter time histories, noise included (time domain identification).

In the last case investigated, the time domain approach was used for identifying the object with IMU and noise included. This case aimed at assessing the wavelet-based approach outcomes in noise presence and again the same settings were used to perform system identification. As no flight parameter decomposition was needed, the estimated model outputs were directly compared to the measured responses. Flight parameter time histories for the identified model are shown in Figure 11, where blue lines denote the measurements and red lines stand for system identification results.

It is clearly visible that the system identification was not accurate in this case. As the projectile was flying at high speed and rotating at a high rate, the errors (due to noise) in the IMU made it impossible to accurately determine the object attitude. Thus, the control sequence was inaccurate (it would be equivalent to launching engines at the wrong radial position). As a result, it led to the estimation inaccuracies for those flight parameters that changed considerably due to the applied control (lateral velocity *v*, vertical velocity *w*, pitch rate *q*, roll rate *r*). For the longitudinal velocity and roll rate, a good match was observed. This is because, due to their large values, they were almost independent of the control.

To solve this issue, it would be possible to directly apply the control history instead of the reference trajectory. However, this approach was beyond the project objectives as the data for estimation can come from multiple missile launches (i.e., from repeated experiments) and during the flight campaign, e.g., the flight conditions can change, resulting in different controls among experiments. On the other hand, the reference trajectory is a predetermined and fixed path.

## 5. Conclusions

In this study, a wavelet-based system identification approach was presented for a spinning projectile controlled with gasodynamic engines. The wavelet coefficients were evaluated for longitudinal and lateral-directional flight parameters and used with the maximum likelihood principle to estimate the aerodynamic coefficients. Additionally, a coherence weighting was used to put more emphasis on the most accurate measurements. This wavelet-based approach was applied for an ideal model and when IMU and noise were taken into account.

For the ideal measurement case, it was possible to obtain high-quality results from the wavelet-based approach and the outcome accuracy was just as high as for the time domain maximum likelihood principle formulation. Due to the flight parameter decomposition, in the wavelet-based approach, more time was required to perform the estimation and it was found that it was not possible to apply this in near real time.

When the IMU and noise were considered, the identified model accuracy slightly decreased when the wavelet-based approach was used. This resulted mainly from longitudinal velocity and roll rate high values, as the control did not produce significant changes in their variations, i.e., the noise had a greater impact. However, the results were still very accurate. When the same set of data was used for time domain maximum likelihood principle estimation, the outcomes were found to be inaccurate due to errors in attitude determination resulting from noise. Thus, for the investigated case, the maximum likelihood wavelet-based approach was found to be superior compared to the time domain formulation.

## Figures and Tables

**Table 1:** Aerodynamic coefficient structure.

Parameter	Formula
$C_{X_{base\;0}}$	4th order polynominal
$C_{X_{base\;\alpha^{2}}}=C_{X_{base\;\beta^{2}}}$	4th order polynominal
$C_{X_{eng\;0}}$	4th order polynominal
$C_{X_{eng\;\alpha^{2}}}=C_{X_{eng\;\beta^{2}}}$	4th order polynominal
$C_{Y_{0}}=C_{Z_{0}}$	0
$C_{Y_{\beta }}=C_{Z_{\alpha }}$	2nd order polynominal
$C_{l_{p_{0}}}$	2nd order polynominal
$C_{l_{p_{\alpha^{2}}}}=C_{l_{p_{\beta^{2}}}}$	2nd order polynominal
$C_{m_{0}}=C_{n_{0}}$	0
$C_{m_{\alpha }}=-C_{n_{\beta }}$	2nd order polynominal

## Data Availability

Not applicable.
